# Supplementary material for: Endometrial Carcinoma: Molecular Cytogenetics and Transcriptomic Profile
Source: Cancers (Basel). 2022 Jul 20;14(14):3536. doi: 10.3390/cancers14143536 (PMC9325179; doi:10.3390/cancers14143536)
Supplement: Supplementary file 1 [file Figure S1.pdf › Figure S1.pdf]

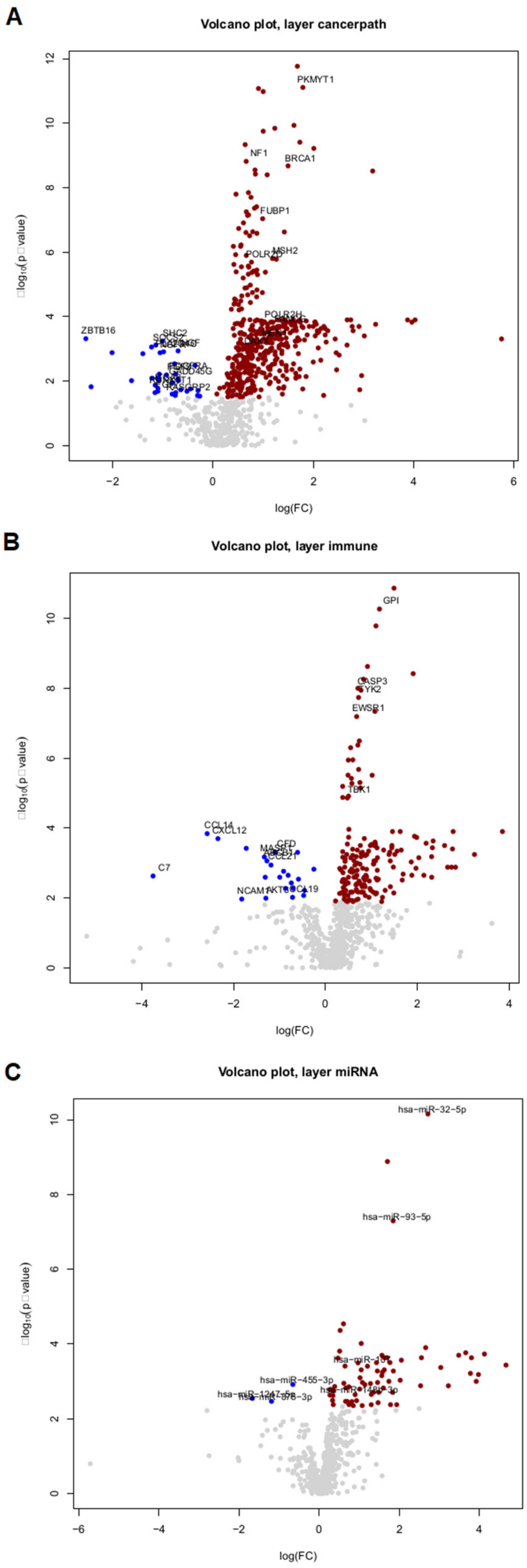

Figure S1. Differential expression (DE) volcano plot for cancer pathway panel (S1A) showing the 10 most upregulated genes, *PKMYT*, *NF1*, *BRCA1*, *FUBP1*, *MSH2*, *POLR2C*, *MEN1*, *POLR2H*, *FANCG*, and *DAXX*, as well as the 13 most downregulated genes, *ZBTB16*, *SHC2*, *SOCS2*, *HGF*, *PLA2G4A*, *NGFR*, *PDGFRA*, *FGF2*, *GADD45G*, *ITGA8*, *RUNX1T*, *FGF7*, and *RASGRP2*.
